# Fulminant Immune Checkpoint Inhibitor‐Related Encephalitis (ICI‐Encephalitis) Presenting Rapidly After First Infusion of Nivolumab: A Case Report

**DOI:** 10.1155/crom/6122845

**Published:** 2026-07-30

**Authors:** Jonathan Markle, Audrey Garza, Alyson Auriemma, Nicholas Rushlow, Mackenzie Eickhoff, Charles Bane

**Affiliations:** ^1^ Department of Internal Medicine, Wright State University Boonshoft School of Medicine, Dayton, Ohio, USA, wright.edu; ^2^ Wright-Patterson Air Force Base, Department of Medicine, Dayton, Ohio, USA, af.mil; ^3^ Department of Graduate Studies, Wright State University Boonshoft School of Medicine, Dayton, Ohio, USA, wright.edu; ^4^ Dayton Physicians LLC, Miami Valley Hospital North, Dayton, Ohio, USA

**Keywords:** immune checkpoint inhibitor-related encephalitis (ICI-encephalitis), immune checkpoint inhibitors (ICIs), neurologic immunotherapy-related adverse event (NirAE), nivolumab, programmed cell death-1 (PD-1)

## Abstract

**Introduction:**

Immune checkpoint inhibitors (ICIs) are increasingly used in oncologic clinical practice, leading to a corresponding rise in immune‐related adverse events (irAEs). In this context, we present a case of fulminant ICI‐associated encephalitis (ICI‐encephalitis) presenting within 24 h after a single administration of nivolumab (a PD‐1 inhibitor), in a patient with recurrent Hodgkin lymphoma.

**Case Presentation:**

After receiving outpatient nivolumab combined with brentuximab (an antibody–drug conjugate), the patient presented with altered mental status, status epilepticus, and abnormal signal in the bilateral temporal lobes as shown on brain magnetic resonance imaging (MRI). Lumbar puncture found pleocytosis and slight elevation in protein; there was no evidence of abnormal autoantibodies or pathogenic infection. Following multidisciplinary consultation, the patient was ultimately diagnosed with ICI‐encephalitis and began on empiric treatment with intravenous methylprednisolone dosed 1 g/day for 5 days, followed by 5 days of intravenous immunoglobulin (IVIG). The patient experienced resolution of electrographic seizures and gradual improvement of mental status toward baseline but had persistent short‐term memory loss and did not experience complete brain MRI recovery. Ultimately, hospice placement was pursued after goals of care discussions with the patient′s spouse, and the patient expired within 1 month of discharge.

**Conclusion:**

This case serves as a safety signal for rapidly presenting ICI‐encephalitis following nivolumab.

## 1. Introduction

Immune checkpoint inhibitors (ICIs) are increasingly used in oncologic clinical practice due to their demonstrated improvement in the prognosis of several cancers [1, 2]. These include programmed cell death‐1 (PD‐1) inhibitors (and inhibitors of its ligands), and cytotoxic T‐lymphocyte‐associated antigen 4 (CTLA‐4) inhibitors. PD‐1 inhibitors restore antitumor immune responses by blocking the interaction between PD‐1 and its ligands, leading to increases in lymphocytic proliferation, T‐cell survival, and inflammatory cytokine production [2]. However, this immunologic reactivation can lead to loss of self‐tolerance against the body’s own cells, leading to complications known as immune‐related adverse events (irAEs). These complications are reported in up to 65% of patients [3]. Neurologic irAEs (NirAEs) are rarer than complications involving most other organ systems but still have a reported incidence of 1%–5% of patients and have the potential to more severely impact quality of life in the cancer patient [1]. NirAEs can occur both centrally (including encephalitis, aseptic meningitis, CNS vasculitis, myelitis, and posterior reversible encephalopathy syndrome) and peripherally (such as neuropathy, myositis, and myasthenia gravis). [4, 5]. Among these events, immune‐related encephalitis has a reported prevalence of less than 1% but, in rare cases, can be fulminant and/or fatal [2]. In this context, we present a case of fulminant ICI‐associated encephalitis (ICI‐encephalitis) presenting within 24 h after a single administration of nivolumab (a PD‐1 inhibitor), in a patient with recurrent Hodgkin lymphoma.

## 2. Case Report/Case Presentation

This report follows the CARE guidelines for case presentations [6]. A preliminary report of this case was previously presented at the 2025 American Society of Hematology Annual Meeting [7]; this report substantially expands details of clinical presentation, treatment, and literature review.

A White male patient in his mid‐70s was diagnosed with recurrent Hodgkin lymphoma in early 2025. His previous cancer history was notable for remote Stage III Hodgkin lymphoma treated with ABVD regimen (doxorubicin, bleomycin, vinblastine, and dacarbazine), and cutaneous low‐grade T‐cell lymphoma treated with topical nitrogen mustard and maintenance phototherapy. Previous medical history was significant for hypothyroidism, hyperlipidemia, essential hypertension, and trigeminal neuralgia. There was no significant surgical history. At the time of presentation, a recent positron emission tomography–computed tomography (PET‐CT) scan revealed disease involving the retroperitoneal and pelvic lymph nodes, without brain involvement.

The patient received his first outpatient infusion of nivolumab and brentuximab without immediate apparent complications, drove himself home, performed computer work in his home office, and then went to his bedroom to rest. That same evening, his spouse found him unresponsive, with foam and blood at his mouth. Emergency medical services were called, who reported at least three visualized seizures on scene without wakefulness in between. He was placed on oxygen using a nonrebreather mask and taken to the hospital, where he was also found to have been incontinent. Per the patient’s spouse, in the preceding several days, there was no complaint of fevers, chills, cough, dyspnea, polydipsia, or nausea, and he was at his neurologic baseline (fully alert and oriented, able to independently undertake his activities of daily living, and able to perform complex tasks such as household budgeting).

Initial vital signs including oxygen saturation were normal, with the patient displaying symptoms of intermittent agitation, poor redirectability, and repetition of nonsensical phrases without aphasia or dysarthria, initially concerning for postictal confusion versus nonconvulsive status epilepticus. He was initially oriented to neither person, place, time, nor situation, with no focal neurologic deficits, normal sensation, normal deep tendon reflexes, normal gait, and no evidence of meningismus. Grossly, the remainder of his physical exam, including cardiopulmonary, abdominal, and extremities, was unremarkable. A chest X‐ray showed no acute cardiopulmonary abnormality. A CT scan of the head, cervical spine, chest, and abdomen/pelvis showed no acute findings. Complete blood count was notable for a white blood cell (WBC) count of 11,400 (reference range [RR] 3.5–10.4 K/*μ*L). A basic metabolic panel was notable for a sodium level of 125 (RR 135–148 mEq/L), decreased from 139 two weeks prior. Urine studies were consistent with SIADH. Initial lactic acid level was 4.5 (RR 0.5–2.2 mmol/L), decreased to 1.5 within 2 h after administration of 1‐L normal saline (NS) in the emergency room (ER). Hepatic function panel, TSH, and random cortisol were within normal limits. Serum osmolality was also within normal limits, but was drawn after administration of the NS. Blood cultures were taken by the ER. High‐sensitivity troponins were initially elevated from 24 (RR < 22 ng/L), ultimately peaking at 63 at 3 h and decreasing 1 h later to 53. EKG showed a normal sinus rhythm without ST/T wave changes compared to a previous one from 2010; thus, troponin elevation was suspected to be demand ischemia. Neurology was consulted, and he was loaded with 4450 mg (60‐mg/kg dose) of levetiracetam due to concern for nonconvulsive status epilepticus, followed by 750‐mg IV twice daily. Hematology–oncology was also consulted. Serial monitoring of metabolic panels was initiated.

**Figure 1:**
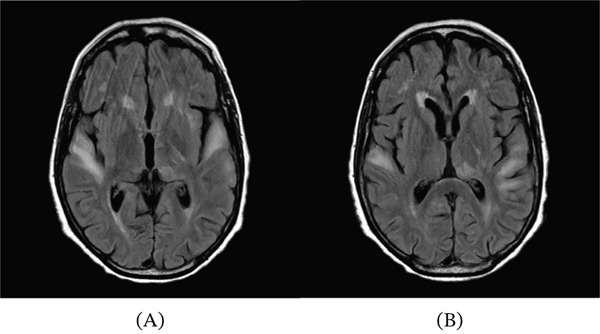
(A, B) Hospital day 2 T2/FLAIR MRI sequences showing new regions of enhancement.

On the first full day of hospitalization, the patient experienced neurologic improvement to orientation of self and intermittently followed simple commands; however, he remained agitated and continued to intermittently repeat phrases. WBC count spontaneously improved to 5100. After 24 h, serum sodium had improved to 135 without further intervention. Brain MRI with and without contrast and continuous electroencephalography (cEEG) were both ordered; however, the patient’s continued agitation precluded these studies from being obtained until Hospital Day 2. He ultimately required transfer to the neurological intensive care unit for dexmedetomidine infusion in order to facilitate diagnostic testing. MRI was notable for new patchy enhancement within the subcortical white matter and cortex of the bilateral temporal operculum and left parietal lobe, with associated non‐expansile hyperintense signal noted on T2/FLAIR sequences (shown in Figure 1A,B) without mass effect. There was no restricted diffusion present to indicate CNS lymphoma, no evidence of acute territorial infarct or intracranial hemorrhage, and no dural venous sinus thrombosis. cEEG was notable for nonconvulsive periodic lateralized epileptiform discharges (PLEDs) in the left posterior quadrant (T5/P3/O1) that evolved three to five times per hour to electrographic seizures, with concomitant moderate continuous generalized slowing. Given emerging clinical concern for ongoing neurologic irAE (NirAE), high‐dose intravenous methylprednisolone (1 g/day for 5 days) was initiated.

By the third full day of hospitalization, the patient’s neurologic status improved to the point where he was intermittently able to converse in short sentences, although he had significant deficits of long and short‐term memory, with continued agitation requiring a bedside safety sitter. He denied symptoms including headache, neck pain, or low back pain when specifically queried. However, given the electrographic seizures despite improvement of hyponatremia and ongoing AED therapy, additional testing was pursued to rule out causes other than immunotherapy‐related toxicity. Serum JC virus with reflex RFL inhibition was negative. Lumbar puncture (LP) with CSF cytology was also performed on this day, and CSF was found clear with glucose of 70 mg/dL (RR 40‐70 mg/dL), three nucleated cells/*μ*L (RR 0‐5/*μ*L), mild lymphocytic predominance (55%), and protein of 81 mg/dL (RR 15–45 mg/dL). Further CSF analyses were negative, including meningitis PCR panel (Table 1), autoimmune encephalopathy panel (Table 2), and paraneoplastic autoantibody panel (Table 3). Bacterial culture and Gram stain of CSF were negative through two further days of hospitalization and then grew *Acinetobacter* species on Hospital Day 6. Broad‐spectrum cefepime 2 g every 8 h was promptly initiated; however, this was strongly felt to represent a bedside contaminant in the setting of neurologic improvement without antibiotics and continued lack of fever, leukocytosis, otherwise discordant CSF studies, and negative blood cultures. Infectious disease was consulted, agreed with this diagnostic logic, and recommended LP by interventional radiology to confirm CSF sterility, and observation for further antibiotics. Repeat LP was performed on Hospital Day 8, which yielded clear CSF with glucose 99 mg/dL, 10 nucleated cells/*μ*L, mild monocytic/macrophage predominance (77%), and protein 90 mg/dL. The meningitis PCR panel was again negative. Additional investigations, including FTA and VDRL were also performed, and resulted negative. CSF Gram stain and culture remained negative through the rest of the hospitalization.

**Table 1:** Meningitis PCR panel.

*Escherichia coli* K1	Not detected
*Haemophilus influenzae*	Not detected
*Listeria monocytogenes*	Not detected
*Neisseria meningitidis*	Not detected
*Streprococcus agalactiae*	Not detected
*Streptococcus pneumoniae*	Not detected
Cytomegalovirus	Not detected
Enterovirus RNA	Not detected
Herpes simplex virus 1	Not detected
Herpes simplex virus 2	Not detected
Human herpesvirus 6	Not detected
Human parechovirus	Not detected
Varicella zoster DNA	Not detected
*Cryptococcus neoformans*/*gattii*	Not detected

**Table 2:** Autoimmune encephalopathy panel.

GAD65 Antibody	Negative
AMPAR1 Ab, CBA	Negative
CASPR2‐IgG CBA, CSF	Negative
CABA‐B‐R Ab CBA, CSF	Not detected
DPPX Ab CBA, CSF	Not detected
GABA‐B‐R Ab CBA, CSF	Not detected
IgLON5 CBA, CSF	Not detected
LGI1‐IgG CBA, CSF	Not detected
NMDA‐R Ab CBA, CSF	Not detected

**Table 3:** Paraneoplastic autoantibody panel.

Amphiphysin	Negative
AGNA‐1	Negative
ANNA‐1, CSF	Negative
ANNA‐2, CSF	Negative
ANNA‐3	Negative
CRMP‐5‐IgG	Negative
PCA‐Tr, CSF	Negative
PCA‐1, CSF	Negative
PCA‐2, CSF	Negative

Despite improvement of mental status from presentation, the patient remained altered from baseline, with administration of the Repeatable Battery for the Assessment of Neurological Status (RBANS) test demonstrating continued significant deficits in the immediate memory, visuospatial/constructional, and delayed memory domains. Formal evaluation by physical and occupational therapy resulted in a recommendation for placement in an extended‐care facility due to impaired safety awareness and short‐term memory deficits in this setting. Patient was treated with 5 days of intravenous immune globulin (IVIG) at 0.4 mg/kg, completed on Hospital Day 13, but experienced no further significant improvement in mental status.

**Figure 2:**
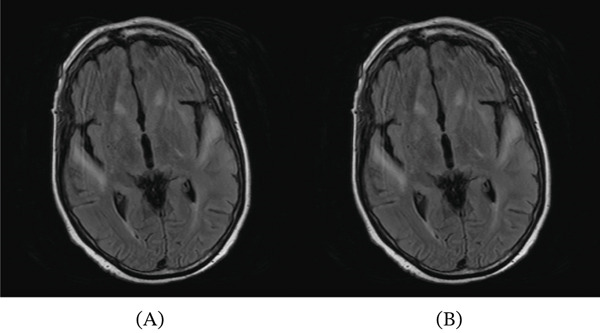
(A, B) Hospital day 14 MRI T2/FLAIR sequences redemonstrating previously visualized enhancement.

Within 1 day of IVIG completion, the patient had a fever for the first time to 101.7°F and was initiated on empiric meningitis coverage again with ceftriaxone, vancomycin, and acyclovir. No leukocytosis or meningismus was noted; however, given previous conflicting CSF culture data, a third LP was performed out of an abundance of caution and again yielded clear CSF with glucose 60 mg/dL, 7 nucleated cells/*μ*L with lymphocytic predominance (77%), and protein 64 mg/dL. Meningitis PCR panel was negative again, and extended testing for West Nile virus RNA PCR was negative. CSF Gram stain and culture remained negative, as with the second culture. The fevers were thus clinically felt to be representative of IVIG‐related infusion reaction, and meningitis coverage was discontinued. The fevers resolved spontaneously without further intervention. A repeat brain MRI with and without contrast was performed on Hospital Day 14, which was degraded by motion, but grossly showed redemonstration of the T2/FLAIR hyperintense signal within the bilateral temporal operculum, left thalamus, and left parietal lobe (shown in Figure 2A,B).

Diagnostic utility of anti‐PD1 antibody testing was discussed with oncologic consultants; however, in light of the patient′s spouse no longer wishing to undergo aggressive treatment measures and pursuing hospice, a tissue sample for testing was not undertaken. The patient was discharged home with home hospice services and passed away within 1 month.

## 3. Discussion

We present a case of rapidly presenting severe encephalitis related to nivolumab, a PD‐1 inhibitor, in which full neurologic recovery was not achieved.

Due to the atypical nature of this case, an exhaustive search of other potential etiologies was undertaken. New metastases to the brain were felt unlikely to be the cause in the setting of the recent PET/CT imaging showing no such lesions, corroborated by inpatient imaging. Imaging also showed no evidence of a new intracranial ischemic event. CSF infection was strongly suspected to be unlikely in the setting of the clinical history, with gram staining, PCR, and other diagnostic testing corroborating this, except for the *Acinetobacter* species, which was felt clinically spurious. The patient did have new mild hyponatremia, but this spontaneously corrected early in the hospitalization, with ongoing evidence of nonconvulsive PLEDs despite serum sodium improvement. Atypical PRES was considered, but the patient did not present severely hypertensive, and also presented with bitemporal FLAIR lesions on MRI.

According to literature, NirAEs have a 23% rate of incomplete neurologic recovery and a 7% fatality rate [8]. In cases that end in fatality, the median time from reported symptom onset to death is a median of 32 days, a time frame consistent with that of our patient [9]. In a multicenter analysis of 21 patients with fatal irAEs, the median time to irAE onset following treatment initiation was 15 days, with the earliest occurring 3 days after treatment initiation [9]. Fourteen of these patients were treated with PD‐1 inhibitors (nine had monotherapy, and five had combined PD‐1/CTLA‐4 blockade). Like our patient, 19 of the patients in the analysis had a history of melanoma or other skin cancers, and the median age was 72 years. Unlike our patient, 9 of 21 had evidence of multisystem irAE. In this population, the median time to onset was 15 days following treatment initiation, with 3 days the fastest reported onset. All patients received high‐dose steroids, with ours receiving steroids faster than the median time of 5 days. Nine subsequently received a second‐line therapy, with four receiving infliximab and five receiving IVIG.

According to the literature, hyperintense signals in the bitemporal regions on T2/FLAIR are the most prevalent MRI finding of ICI‐associated encephalitis. Our patient demonstrated these characteristic MRI findings. [5].

There are several unique points raised from this case that remain without satisfactory explanation. Although other cases of rapidly presenting ICI‐encephalitis have been reported, our patient had symptomatic onset within 24 h, grossly discordant with the median onset of all NirAEs of 6–8 weeks [10, 11, 12]. Our patient also experienced this complication after the first treatment cycle, in contrast to the median of 3 to 5.5 cycles reported by other researchers[5, 13]. Our patient did have a previous history of radiotherapy for his cutaneous T‐cell lymphoma; however, the patient had not received any radiotherapy within the 90 days prior to the NirAE, and a large meta‐analysis of patients with irAEs has found comparable rates of AEs in patients who did and did not receive radiotherapy [14]. It is also notable that our patient received brentuximab, an antibody–drug conjugate targeting CD30(+) cells; none of the patients in the multicenter fatal irAE analysis received a conjugate with the PD‐1 inhibitor, but if there is any relation to this and the unique presentation of our case is uncertain. Finally, a limitation of this case is that the anti‐Ma2 antibody (an antibody with a reported association with ICI‐encephalitis) was unable to be tested for, as it is not included in our facility′s autoantibody panel [15].

In conclusion, we report a case of nivolumab‐associated fulminant ICI‐encephalitis, with several atypical features compared to previously described literature. With the use of ICIs in patients with malignant tumors becoming increasingly common, reporting of such cases is crucial for future clinician awareness.

## Author Contributions

**Jonathan Markle:** regulatory, literature review, writing/editing/review of manuscript, submission of manuscript. **Audrey Garza:** literature review, writing/editing/review of manuscript. **Alyson Auriemma:** literature review, writing/editing/review of manuscript. **Nicholas Rushlow:** literature review, writing/editing/review of manuscript. **Mackenzie Eickhoff:** literature review, writing/editing/review of manuscript. **Charles Bane:** writing/editing/review of manuscript.

## Funding

No funding was received for this manuscript.

## Ethics Statement

As this case report was of a single individual, exemption from Institutional Review Board (IRB) review was given by the Department of Clinical Research at Premier Health.

## Consent

Informed consent for publication of the case with accompanying images was obtained from the legal next of kin (spouse) of the patient, as they were unable to consent in the setting of illness. The signed and dated informed consent was filed with the Department of Clinical Research at Premier Health, with a copy given to the next of kin.

## Conflicts of Interest

J.M., A.G., A.A., N.R., and M.E. have no conflicts of interest to disclose. C.B. is the outpatient oncologist for the patient described in this report.

## Data Availability

All data generated or analyzed during the report of this case are included in this article. Further enquiries can be directed to the corresponding authors.
